# ToLCNDV-ES infection in tomato is enhanced by TYLCV: Evidence from field survey and agroinoculation

**DOI:** 10.3389/fmicb.2022.954460

**Published:** 2022-11-08

**Authors:** Thuy Thi Bich Vo, Elisa Troiano, Aamir Lal, Phuong Thi Hoang, Eui-Joon Kil, Sukchan Lee, Giuseppe Parrella

**Affiliations:** ^1^Department of Integrative Biotechnology, Sungkyunkwan University, Suwon, South Korea; ^2^Institute for Sustainable Plant Protection, National Research Council (IPSP-CNR), Portici, Italy; ^3^Department of Plant Medicals, Andong National University, Andong, South Korea

**Keywords:** geminiviruses, tomato, virus complementation, infectious clones, TYLCV, ToLCNDV

## Abstract

The tomato leaf curl New Delhi virus (ToLCNDV), a bipartite begomovirus (family *Geminiviridae*), poses a significant threat to various horticultural crops in many Asian and Mediterranean countries. Since its identification, the Asian strain of this virus has exhibited severe infectivity and caused high yield loss in tomato and cucurbit production in the Indian subcontinent and other parts of Asia. ToLCNDV-ES, a different strain of ToLCNDV, emerged recently in the Mediterranean Basin and caused significant outbreaks in *Cucurbitaceae* species but has shown low adaptation to tomatoes. In a field survey, tomato plants infected with this Mediterranean strain were not discovered. Nevertheless, the same field survey revealed that ToLCNDV-ES occurred in natural double infection with tomato yellow leaf curl virus (TYLCV) in tomato plants with an infection ratio of up to 50%. Moreover, results obtained from experiments where tomato plants agro-inoculated simultaneously with infectious clones of ToLCNDV-ES and TYLCV showed that ToLCNDV-ES was detected in tomatoes while synergized with TYLCV with infection ratios similar to those found under field conditions. Quantitative PCR data indicated the highest amount of ToLCNDV in co-infected plants and no significant change in TYLCV titers among the different mixed infections. Moreover, it was ascertained that not all begomoviruses can enhance the infectivity of Mediterranean ToLCNDV isolates in tomato plants. Our study reports a new finding regarding the ToLCNDV-ES response in tomato while synergized with TYLCV with evidence from both field and laboratory conditions.

## Introduction

*Begomovirus*, the largest genus in the family *Geminiviridae*, with 445 species, is a major cause of disease in numerous crops worldwide (Varma et al., 2011). Some begomoviruses are monopartite, with only one DNA component, or bipartite with two DNAs designated as DNA A and DNA B (Zerbini et al., 2017). Begomoviruses were mainly transmitted by the whitefly *Bemisia tabaci*, however, recently seed transmission was also reported in several species including sweet potato leaf curl virus (Kim et al., 2015), tomato yellow leaf curl virus (TYLCV; Kil et al., 2016, 2017, 2018) despite some controversy (Pérez-Padilla et al., 2020), bitter gourd yellow mosaic virus (Manivannan et al., 2019), tomato leaf curl New Delhi virus (ToLCNDV; Sangeetha et al., 2018; Kil et al., 2020), dolichos yellow mosaic virus (Suruthi et al., 2018) and pepper yellow leaf curl Indonesia virus (Fadhila et al., 2020). Begomovirus infects abroad range of monocotyledonous and dicotyledonous plants, of which tomatoes are the most permissive hosts, the large-scale infection of which can result in severe economic losses (Hanssen et al., 2010).

ToLCNDV is an economically important member of the *Begomovirus* genus and has a bipartite genome structure (Moriones et al., 2017). ToLCNDV was initially reported in tomatoes in India approximately 25 years ago (Padidam et al., 1995) and then identified in cucurbits in the Mediterranean Basin in 2012 (Juárez et al., 2014; Mnari-Hattab et al., 2015; Panno et al., 2016; Parrella et al., 2018; Orfanidou et al., 2019). Along with *Solanaceae* and *Cucurbitaceae* (its host species), this virus has been reported to infect plants belonging to *Malvaceae, Fabaceae, Phyllanthaceae, Papaveraceae* and *Euphorbiaceae* families (Srivastava et al., 2016; Jamil et al., 2017; Venkataravanappa et al., 2018; Sharma et al., 2021). It is as a serious hindrance in the production of various crops in different countries because of its rapid infectivity and the extent of outbreaks it can cause.

Tomatoes are one of the most important crops with a high economic value, constituting up to 72% of the value of fresh vegetables produced worldwide (Hanssen et al., 2010). Its production is affected by numerous diseases, approximately 50% of which are caused by plant viruses. Databases revealed that tomatoes have the highest infection recorded for any plant; at least 312 viruses, satellite viruses, and viroid species are known to be able to infect them (Rivarez et al., 2021). The occurrence of TYLCV is a prevalent limiting factor for tomato production in many regions, including North Africa, East Asia, the Mediterranean, the Caribbean, and America (Mabvakure et al., 2016).

The isolates of ToLCNDV from Asian and Mediterranean countries (ToLCNDV-ES) are categorized as two different strains based on full-length sequences and performed distinct adaptations in tomato plants. The ToLCNDV Asian strain causes significant diseases in tomato with severe symptoms such as stunted or dwarfed growth; leaflets being curled upwards and inwards, slightly chlorotic and yellowish displays, crumpling, and mosaic/mottling (Singh et al., 2015). Despite this, the ToLCNDV-ES strains are mainly adapted to cucurbits and infect tomatoes with great difficulty; they have shown low field incidence in this plant (Yamamoto et al., 2021). In Spain, latent infection of ToLCNDV-ES (Fortes et al., 2016) and symptoms of slight leaf chlorotic mosaic (Ruiz et al., 2017), have been reported in tomato. Attenuated symptoms are correlated with low levels of ToLCNDV-ES accumulation in tomatoes, as opposed to the titers detectable in zucchini, especially with respect to the DNA B component. In addition, the transmission efficiency mediated by *B. tabaci* was significantly higher in zucchini (96%) than in tomatoes (2%). Overall, these features indicated that tomato is a less or not permissive host for ToLCNDV-ES (Simón et al., 2018). In fact, until now, after 7 years of ToLCNDV-ES monitoring in solanaceous and cucurbits crops (URCOFI project), this virus was never detected in tomato crops of continental Italy (in particular in Campania and Lazio regions), where the Italian subgroup I of ToLCNDV-ES isolates have been described so far (Panno et al., 2019). Interestingly, based on CP sequences, the Italian ToLCNDV-ES isolates are divided in two main subgroups: the subgroup I, grouping only Italian isolates (from Campania and Lazio regions) and the subgroup II, in which are grouped Spanish and Italian isolates, the latter only those from Sicily (Panno et al., 2019).

In a previous study, an infectious clone of ToLCNDV-ES that was obtained from a ToLCNDV-infected pumpkin plant, identified in Campania region (Southern continental Italy), was successfully constructed and its biological features were compared with that of an Asian strain, especially in cucurbits (Parrella et al., 2018; Vo et al., 2022).

In this study, we tested the infectivity of the ToLCNDV-ES Italian isolate *via* agroinoculation in different tomato cultivars using its infectious clone and demonstrated that this isolate was able to infect tomato plants (with a low viral titer) only when co-inoculated with TYLCV. These results confirmed previous observations made during a field survey in 2019–2020 in Italy of tomato crops that were naturally exposed to TYLCV and ToLCNDV-ES infections that were mediated by *B. tabaci*. The results showed that no ToLCNDV-ES infection was found in tomatoes, which is slightly different from the spread of ToLCNDV-ES isolates in Spain. The outcomes of inoculations of various mixes were also examined using infectious clones to determine whether TYLCV enhanced ToLCNDV-ES infection in tomatoes. Polymerase chain reaction (PCR) and quantitative PCR (qPCR) data indicated that ToLCNDV was detected, at a low ratio, only when co-inoculated with TYLCV. Other tomato-infected begomoviruses included tomato yellow leaf curl Kanchanaburi virus (TYLCKaV) and tomato leaf curl Joydebpur virus (ToLCJoV) did not induce ToLCNDV infection as TYLCV did. Our study highlights a new finding related to ToLCNDV-ES pathogenicity in tomato associated with TYLCV co-infection under both field and laboratory conditions. Apart from the findings obtained, our study prompts the identification of the key factors of ToLCNDV-ES emergence in tomatoes in future research.

## Materials and methods

### Field survey

**Figure 1:**
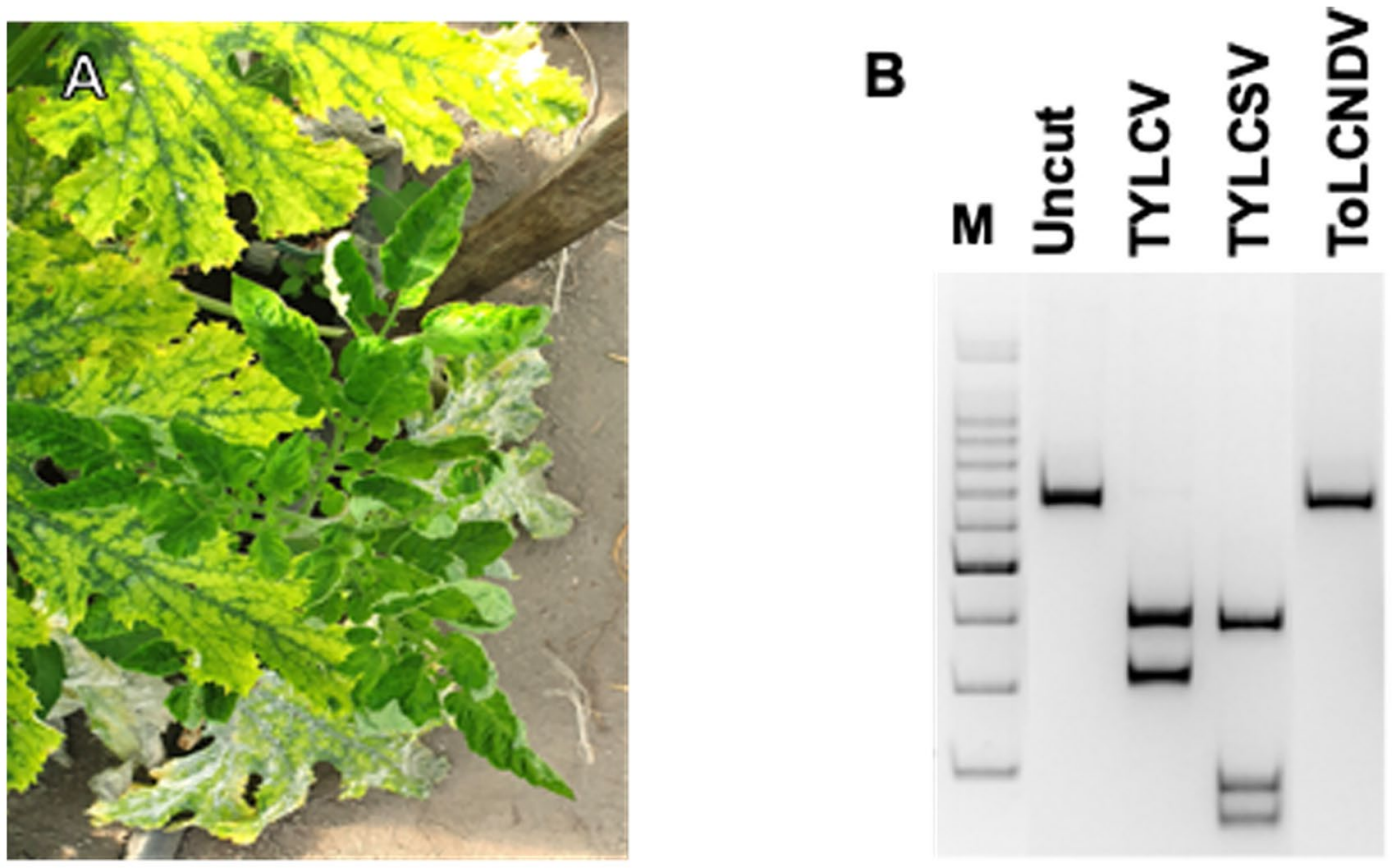
Virus detection on tomato plants: **(A)** symptomatic tomato plant, grown spontaneously among plants of a zucchini crop, also showing geminivirus symptoms; **(B)** PCR-RFLP based on *Ava*II digestions of the amplicons obtained by PCR with Gem1f/Gem2r primers on DNA extracted from tomato plants individually infected by the three geminiviruses.

A total of 71 tomato leaf samples, regardless of whether the plants showed symptoms or not, and 40 zucchini symptomatic leaf samples, were collected in 2019 and 2020 from a greenhouse (sampling site coordinates: 40°45 N, 14°25′E) in which tomato and zucchini were intercropped during the spring–summer period (Table 1). The first full expanded leaves from the apex of both tomato and zucchini plants were sampled. In particular, 48 symptomatic and 23 symptomless tomato samples were collected (Table 1). Some tomato samples were taken from plants born spontaneously among the zucchini plants, most likely born from seeds originating from fallen fruits of the previous crop (Table 1 and Figure 1A). Samples were kept in a refrigerated field bag, transported the same day to the laboratory and immediately processed. The quantity necessary for DNA extraction was taken from each sample (see section “DNA extraction and PCR analysis”), and the remainder was dried in calcium chloride and stored at 5°C, with the aim of returning to the same sample for further investigation if it was necessary.

**Table 1:** Synthesis of the results obtained performing the PCR-RFLP on the DNA extracted from 71 tomato plants in cultivation or spontaneously born among zucchini plants.

Sample	Variety/type	Date of sampling	Symptoms	PCR	RFLP results
1	Jama	04/11/19	Yes	+	TYLCV+ToLCNDV
2	Jama	04/11/19	Yes	+	TYLCV+ToLCNDV
3	Jama	04/11/19	No	−	n/a
4	Jama	04/11/19	Yes	+	TYLCV
5	Jama	04/11/19	Yes	+	TYLCV
6	Jama	02/11/20	Yes	+	TYLCV+ToLCNDV
7	Jama	02/11/20	Yes	+	TYLCV+ToLCNDV
8	Jama	02/11/20	Yes	+	TYLCV+ToLCNDV
9	Jama	02/11/20	Yes	+	TYLCV+ToLCNDV
10	Jama	02/11/20	Yes	+	TYLCV
11	Adelante	04/11/19	Yes	+	TYLCV+ToLCNDV
12	Adelante	04/11/19	Yes	+	TYLCV+ToLCNDV
13	Adelante	04/11/19	Yes	+	TYLCV+ToLCNDV
14	Adelante	04/11/19	Yes	+	TYLCV
15	Adelante	02/11/20	Yes	+	TYLCV+ToLCNDV
16	Adelante	02/11/20	Yes	+	TYLCV
17	Adelante	02/11/20	Yes	+	TYLCV
18	Adelante	02/11/20	Yes	+	TYLCV
19	Adelante	02/11/20	Yes	+	TYLCV+ToLCNDV
20	Adelante	02/11/20	Yes	+	TYLCV
21	spontaneous	02/11/20	Yes	+	TYLCV
22	spontaneous	02/11/20	Yes	+	TYLCV+ToLCNDV
23	spontaneous	02/11/20	Yes	+	TYLCV+ToLCNDV
24	spontaneous	02/11/20	Yes	+	TYLCV
25	spontaneous	02/11/20	Yes	+	TYLCV+ToLCNDV
26	spontaneous	02/11/20	Yes	+	TYLCV+ToLCNDV
27	spontaneous	02/11/20	Yes	+	TYLCV+ToLCNDV
28	spontaneous	10/11/20	Yes	+	TYLCV
29	spontaneous	10/11/20	No	−	n/a
30	spontaneous	10/11/20	No	−	n/a
31	spontaneous	10/11/20	No	−	n/a
32	spontaneous	10/11/20	Yes	+	TYLCV
33	spontaneous	10/11/20	Yes	+	TYLCV
34	spontaneous	10/11/20	No	−	n/a
35	San Marzano	10/11/20	Yes	+	TYLCV
36	San Marzano	10/11/20	Yes	+	TYLCV
37	San Marzano	10/11/20	Yes	+	TYLCV
38	San Marzano	10/11/20	No	−	n/a
39	San Marzano	10/11/20	No	−	n/a
40	San Marzano	10/11/20	Yes	+	TYLCV+ToLCNDV
41	spontaneous	19/11/20	No	−	n/a
42	spontaneous	19/11/20	Yes	+	TYLCV
43	spontaneous	19/11/20	Yes	+	TYLCV
44	spontaneous	19/11/20	No	−	n/a
45	spontaneous	19/11/20	Yes	+	TYLCV
46	spontaneous	19/11/20	Yes	+	TYLCV
47	spontaneous	19/11/20	Yes	+	TYLCV
48	spontaneous	19/11/20	Yes	+	TYLCV+ToLCNDV
49	spontaneous	19/11/20	Yes	+	TYLCV+ToLCNDV
50	spontaneous	19/11/20	Yes	+	TYLCV+ToLCNDV
51	spontaneous	19/11/20	Yes	+	TYLCV+ToLCNDV
52	spontaneous	19/11/20	Yes	+	TYLCV+ToLCNDV
53	spontaneous	19/11/20	Yes	+	TYLCV+ToLCNDV
54	spontaneous	19/11/20	No	−	n/a
55	spontaneous	19/11/20	No	−	n/a
56	spontaneous	19/11/20	No	−	n/a
57	spontaneous	19/11/20	No	−	n/a
58	spontaneous	19/11/20	Yes	+	TYLCV+ToLCNDV
59	spontaneous	19/11/20	No	−	n/a
60	spontaneous	19/11/20	No	−	n/a
61	spontaneous	19/11/20	No	−	n/a
62	spontaneous	19/11/20	Yes	+	TYLCV
63	spontaneous	19/11/20	No	−	n/a
64	spontaneous	19/11/20	Yes	+	TYLCV+ToLCNDV
65	spontaneous	19/11/20	No	−	n/a
66	spontaneous	19/11/20	Yes	+	TYLCV
67	spontaneous	19/11/20	No	−	n/a
68	spontaneous	19/11/20	No	−	n/a
69	spontaneous	19/11/20	No	−	n/a
70	spontaneous	19/11/20	No	−	n/a
71	spontaneous	19/11/20	No	−	n/a

Restriction profiles of each sample are shown in Figure 1. Underlined virus acronyms indicate virus samples that displayed faint bands in acrylamide gels and for which infection was further confirmed by specific qPCR (not shown). n/a=not applicable.

In the sampling location, TYLCV has been prevalent there since 2003 when it was first identified on tomatoes both by itself and in mixed infections with tomato yellow leaf curl Sardinia virus (TYLCSV; Parrella et al., 2006). In the following years, TYLCV was constantly found on this site on tomato cultivations, especially in the summer-autumn period. Instead, ToLCNDV-ES has been identified in the same site on cucurbits since 2017 (Panno et al., 2016; Parrella et al., 2018). During the survey of both crops, tomato and zucchini, showed obvious symptoms of geminivirus infection from early summer, coinciding with the increase in the population of *B. tabaci* (Figure 1A).

### DNA extraction and PCR analysis

Total DNA was extracted from leaf tissue samples using the E.Z.N.A. Plant DNA kit (Omega Bio-tek, Norcross, GA). DNA concentration was determined with the BioSpectrometer basic (Eppendorf, United States), using the μCuvette G1.0 (Eppendorf, United States), and adjusted to a final concentration of about 50 ng/μL. Each DNA samples was then used for PCR analysis to detect TYLCV and ToLCNDV-ES.

Primers were designed manually from a multi alignment including the nucleotide sequences of the coat protein (CP) gene from representative Italian TYLCSV, TYLCV and ToLCNDV-ES isolates deposited in the National Center for Biotechnology Information (NCBI). The degenerated primer pair Gem1f (5’-ACKCCCGYMTCGAAGGTWCG-3′) and Gem2r (5’-GCATGMGTACAKGCCATATAC-3′) was selected in order to amplify a fragment of about 680 bp, comprising about 87% of the CP Open Reading Frame (ORF) of the three geminiviruses being studied. These primers were derived from primers TYCP1(+) and TYCP2(−), used previously for typing tomato yellow leaf curl viruses spreading in tomato crops in southern Italy (Parrella et al., 2005, 2006), in which, based on multi-alignment of Italian isolates of TYLCV, TYLCSV and ToLCNDV-ES of the CP genes, some additional degenerations were introduced in order to amplify also the Italian isolates of ToLCNDV-ES. Before use these primers for extensive field surveys, they were checked several times to detect the above geminiviruses by PCR end-point both in plants with simple/mixed infections and by using plasmids in which the CPs ORF of the three geminiviruses were cloned (not shown).

PCR amplification was performed in volumes of 25 μl, each containing 2.5 μl of the template genomic DNA, 12.5 pmol of each primer, 12.5 μl of GoTaqGreen Master Mix (Promega, United States) and 9.5 μl of sterile distilled water, using a Mastercycler Nexus X2 (Eppendorf, United States) thermocycler, under the following conditions: 3 min at 94°C as pre-denaturation, thermal cycling for 35 cycles (50 s at 94°C, 50 s at 55°C and 1 min at 72°C), and 10 min at 72°C as final extension. PCR products were visualized on 1.2% agarose gel and stained with ethidium bromide.

### Geminivirus identification in tomato and *Bemisia tabaci* by restriction fragment length polymorphism of PCR products

The sequences of amplicons obtained, from tomato showing TYLCD syndrome, with Gem1f/Gem2r primer pairs corresponding with the geminiviruses TYLCSV, TYLCV, and ToLCNDV-ES (Italian isolates) were analyzed with MacVector software version 17.5.6 (Accelrys, Inc., United States) to obtain restriction maps, and a restriction fragment length polymorphism (RFLP)-based assay was developed. Under the conditions recommended by the supplier, 3 μl of purified PCR product was digested in a 10 μl mixture consisting of 5 U of *Ava*II (EURx, Gdańsk, Poland), 0.2 μl of 100× bovine serum albumin (BSA), 1 μl of the appropriate 10x restriction buffer (ONE buffer) and 5 μl of sterile distilled water. The digestion mixture was incubated at 37°C for 2 h, followed by heat-inactivation at 65°C for 20 min. The digested products were separated by 6% polyacrylamide gel electrophoresis (PAGE) and visualized by ethidium bromide staining. The *B. tabaci* specimens were checked for the presence of the three geminiviruses, following the same method described above for symptomatic tomatoes.

### Identification of *Bemisia tabaci* genotype

A total of 10 *B. tabaci* adults were sampled from symptomatic tomato plants. Specimens were stored at −20°C in 95% ethanol until molecular proceedings were carried out. *B. tabaci* samples were genotyped using the method described previously (Parrella et al., 2012; Bertin et al., 2018, 2021). This method allows the identification of all the *B. tabaci* genotypes described so far in the Mediterranean basin by PCR-RFLP of the amplicon corresponding to the partial sequence of the cytochrome oxidase I gene (COI; Frohlich et al., 1999).

### Construction of ToLCNDV and TYLCV infectious clones

Infectious clones of TYLCV and ToLCNDV-ES were obtained by constructing a tandem repeat fragment of the full-length viral DNA as previous described (Urbino et al., 2008).

To generate infectious clones of TYLCV, each partial fragment of approximately 1.6 kb and 1.4 kb was amplified by the newly designed primer sets (Supplementary Table S1) based on the genome of TYLCV isolate Goseong (Accession number: JN680149), and cloned into the pGEM T-easy vector (Promega, United States) to yield pGEM-TYLCV-0.6 mer/-0.5 mer. The cloned fragments were digested with *Sal*I*, Sph*I and *Bgl*II, and then ligated into the pCAMBIA 1303 vector to obtain the pCAMBIA-TYLCV-1.1 mer construct. The recombinant pCAM-TYLCV-1.1 mer was the used to transform the *Agrobacterium tumefaciens* strain GV3101 by electroporation. By using the same approach described, a ToLCNDV-ES infectious clone was successfully constructed in a previous study by using a ToLCNDV-ES Italian isolate from pumpkin (Parrella et al., 2018; Vo et al., 2022).

### Agroinoculation and symptoms observation

For the single infection, *A. tumefaciens* containing each recombinant plasmid were grown in 20 ml of Luria-Bertani (LB) broth media supplemented with kanamycin (50 μg/ml), rifampicin (50 μg/ml), and gentamycin (50 μg/ml) for 24 h at 28°C and 200 rpm until it reached an optical density (OD) of 1.0 at 600 nm. Liquid culture were then inoculated into tomato through pinpricking method. For the co-inoculation test, cell cultures of *A. tumefaciens* that had been mixed separately with the two different infectious clones were centrifuged, and the pellet was plated at a ratio of 1:1 on LB medium with pH 5.7 for 2 h and contained 200 mm acetosyringone, 10 mm MgCl_2,_ and 10 mm MES. The activated *A. tumefaciens* cell culture was then used for agroinoculation of 3-week-old tomato plants *via* pinpricking of the main apical shoot (Sahu et al., 2010).

A total of 5 Italian cultivars of tomato (San Pedro, Roma VF, Principe Borghese, San Marzano 2, Sorrento), one Korean cultivar (Seogwang), one TYLCV-resistant tomato cultivar (Bacchus), and the Moneymaker tomato lines were used in the single and co-inoculation tests. For each cultivar/line, 10 plants were agroinoculated and maintained in a growth chamber (16/8 h light/dark periods, at 22–28°C). All inoculated plants were observed daily, up to 3 weeks post-inoculation. The presence of ToLCNDV-ES and TYLCV in singly or double-inoculated plants was assessed by a PCR with specie-specific primer sets (Supplementary Table S1), and the symptomatology was recorded with a digital camera.

### Determination of viral titer by qPCR

To determine the viral titer, qPCR was conducted on agroinoculated plants 21 days post-inoculation (dpi). Total DNA from the three plants was extracted using the DNeasy Plant Mini Kit (Qiagen, United States) and quantified using an Epoch microplate spectrophotometer (Biotek, Seoul, Korea). Equal amounts of genomic DNA (20 ng) were used as templates in the qPCR containing 5 μl of TB Green® Premix Ex Taq™ II (Tli RNAseH Plus; TaKaRa Bio, Japan), 1 μl of both TYLCV and ToLCNDV-ES qPCR primer sets (Supplementary Table S1), and sterile water, resulting in a final volume of 15 μl. The elongation factor-1α gene (EF-1α) was used as an internal control for the normalization of the amplification reactions (Wang et al., 2020) and each reaction was replicated three times.

The reaction conditions were as follows: pre-denaturation at 95°C for 10 min followed by 30 cycles (for TYLCV) and 35 cycles (for ToLCNDV-ES) of a denaturation step at 95°C for 10 s, an annealing step (at 58°C for TYLCV and, 60°C for ToLCNDV-ES) for 15 s, and an extension step at 72°C for 20 s. Data analyses were performed using the 2^−ΔΔCt^ method (Livak and Schmittgen, 2001). Statistical analyses were performed using the t-test using GraphPad Prism (GraphPad Software, United States).

## Results

### Identification of TYLCSV, TYLCV and ToLCNDV-ES by PCR-RFLP

**Figure 2:**
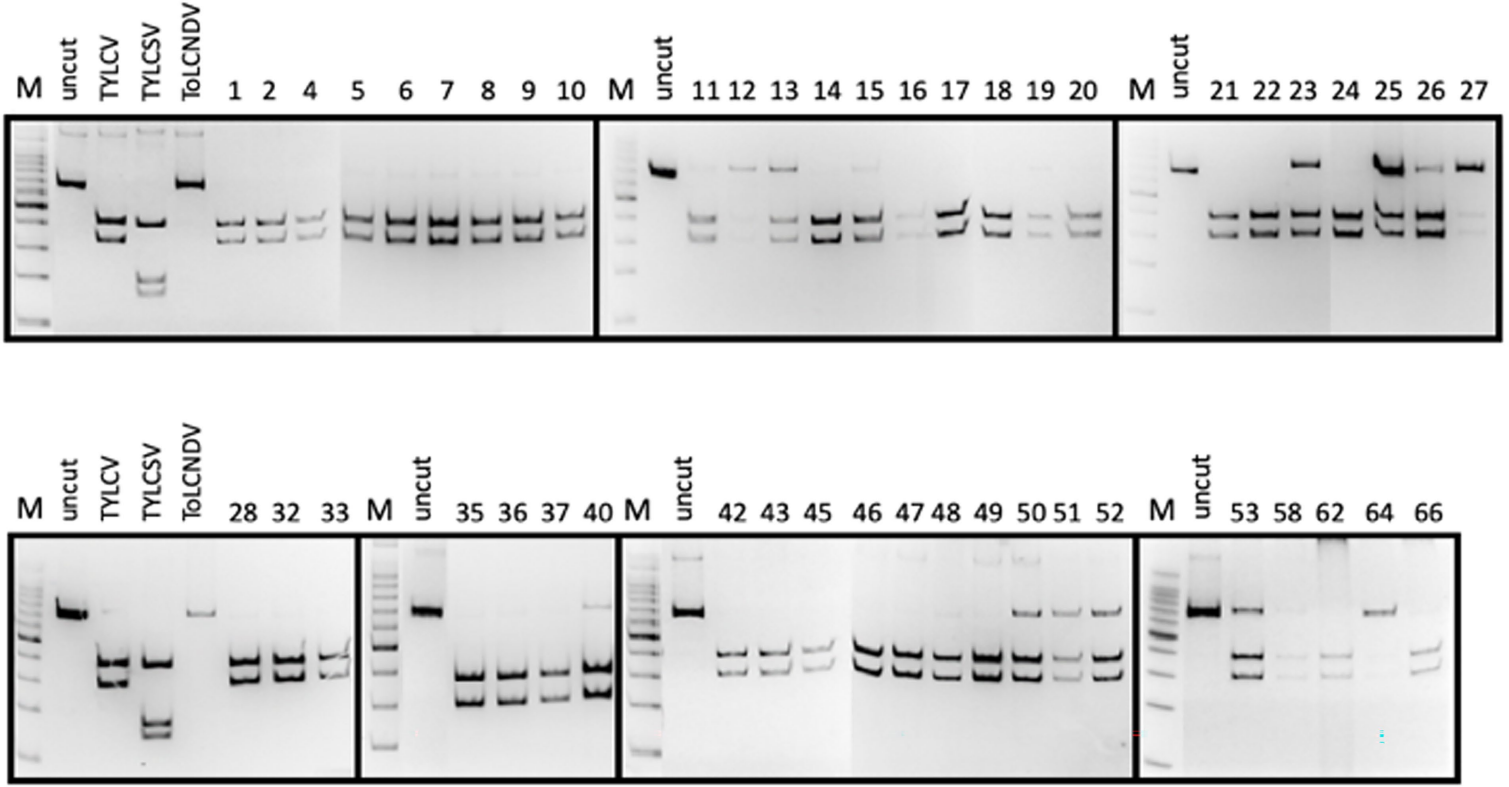
PCR-RFLP profiles obtained from each tomato plant sampled and checked for the presence of TYLC, TYLCSV, and ToLCNDV. The presence of viruses in samples showing weak fragments in acrylamide gels was further confirmed by specific qPCR (not shown).

**Figure 3:**
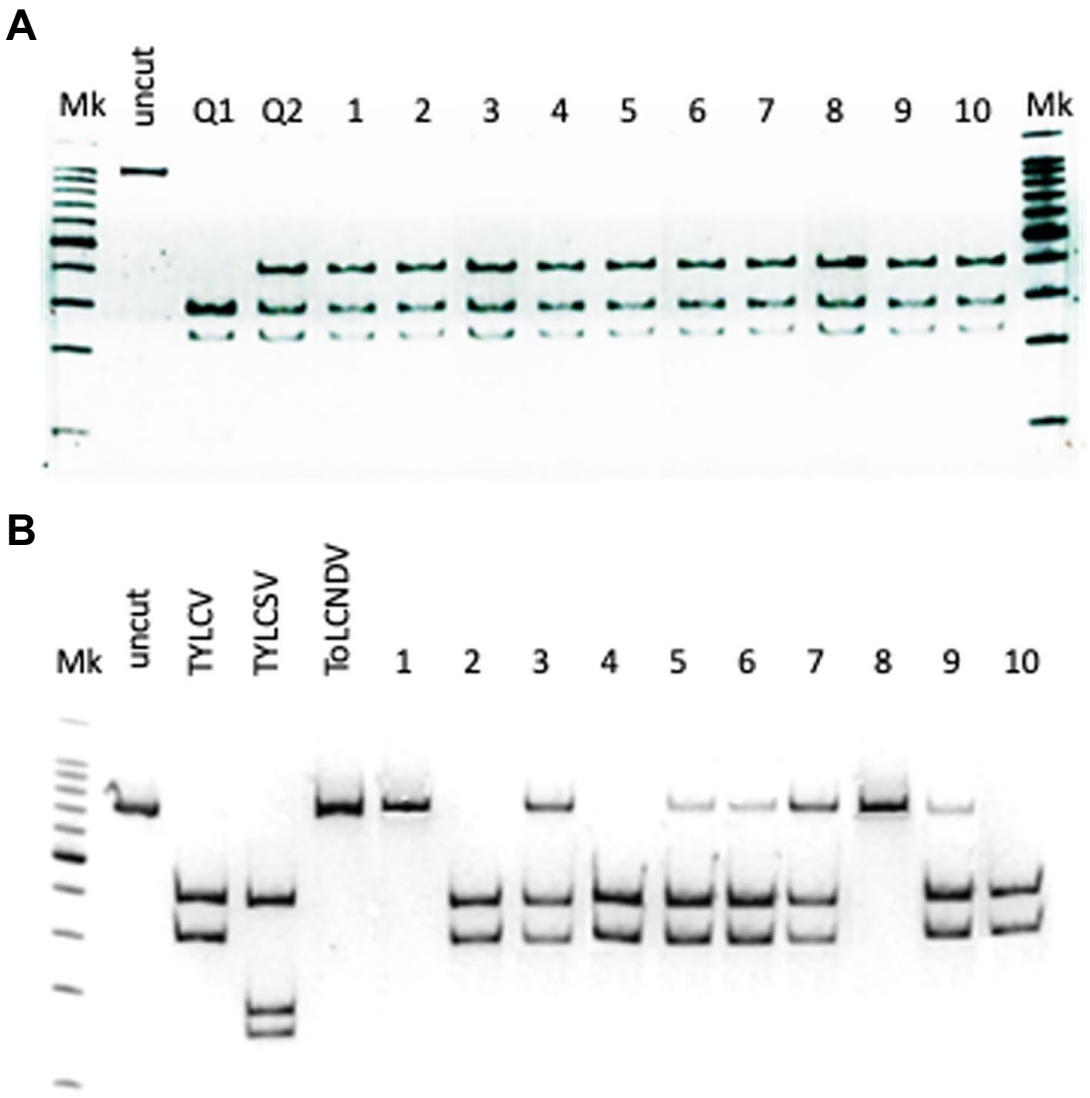
**(A)** Identification of the genotype in 10 *Bemisia tabaci* specimens sampled on tomato showing TYLCD, based on *Apo*I restriction of partial COI gene amplicons; lane Mk: ladder; lane uncut: undigested amplicon; lane Q1: restriction profile of the Q1 mitotype control; lane Q2: restriction profile of the Q2 mitotype control; lanes 1–10: restriction profiles of 10 *B. tabaci* specimens sampled, based on *Ava*II restriction of the amplicons corresponding to the partial CP of the three geminiviruses. **(B)** Identification of geminiviruses in the 10 *B. tabaci* sampled; lane Mk: ladder; lane uncut: undigested amplicon; lane TYLCV: control TYLCV amplicon; lane TYLCSV: control TYLCSV amplicon; lane ToLCNDV: control ToLCNDV amplicon; lanes 1–10: restriction profiles of the 10 *B. tabaci* specimens.

The PCR results indicated that Gem1f/Gem2r primers were able to amplify each of the corresponding genomic regions of the three geminiviruses, giving a robust single amplicon of approximately 680 bp (not shown). Nevertheless, multi-alignment of the amplified genomic portions of the three viruses, encompassed by Gem1f/Gem2r primers, indicated that putative amplicons were slightly different, depending on the virus. The precise length of each amplicon was 680 bp for TYLCV, 677 bp for TYLCSV, and 674 bp for ToLCNDV-ES, corresponding to 87% of the CP ORF in all cases. However, these differences in amplicon lengths were not sufficient to differentiate the three geminiviruses, even when using PAGE. Instead, digestion of the amplicons with *Ava*II endonuclease provided a specific restriction profile for each of the Italian isolates of the three viruses, allowing them to be easily distinguished in both single and mixed infections (Figure 1B). This method was adopted to screen both tomato and *B. tabaci* samples, in order to identify the viral species associated to tomato (Figure 2) and vector (Figure 3B) during field survey.

### Identification of *Bemisia tabaci* genotype and detection of geminiviruses in the vector

Based on the restriction profiles obtained after the digestion of the amplicon corresponding to the partial sequence of the COI gene (Parrella et al., 2012), all specimens analyzed belonged to the mitotype Q2 of the *B. tabaci* MED (Mediterranean) genotype (Figure 3A).

In the same specimens, TYLCV and ToLCNDV were also detected in all possible combination. ToLCNDV was detected alone in two specimens, while TYLCV in three specimens. The rest of the samples (50% of the specimens) showed the presence of both geminiviruses (Figure 3B).

### ToLCNDV-ES infection was associated with TYLCV in field survey

The PCR-RFLP method described above was applied to check tomato samples for geminivirus infection during the field survey. The results are presented in Table 1 and Figure 2. A strong correlation was found between symptomatic plants and the presence of at least one geminivirus. TYLCSV was never found to be associated with symptomatic plants, whereas 48% of the tomato plants were individually infected with TYLCV (23 of 48 plants tested positive by PCR with Gem1f/Gem2r primers) and about 52% (25 plants out of 48 plants that had tested positive) were double-infected with both TYLCV and ToLCNDV-ES. No plant was infected by ToLCNDV-ES alone, and the remaining asymptomatic plants (23 out of 71 plants tested) were virus-free. Based on these results, ToLCNDV-ES infection in tomato was associated with the simultaneous presence of TYLCV. In some cases, when restriction digestion showed the presence of weak amplicons of TYLCV/ToLCNDV-ES in putative double-infected plants, the presence of both viruses was confirmed by qPCR as described previously (see section “Determination of viral titer by qPCR”).

### Coinoculation of TYLCV and ToLCNDV infection clones

**Figure 4:**
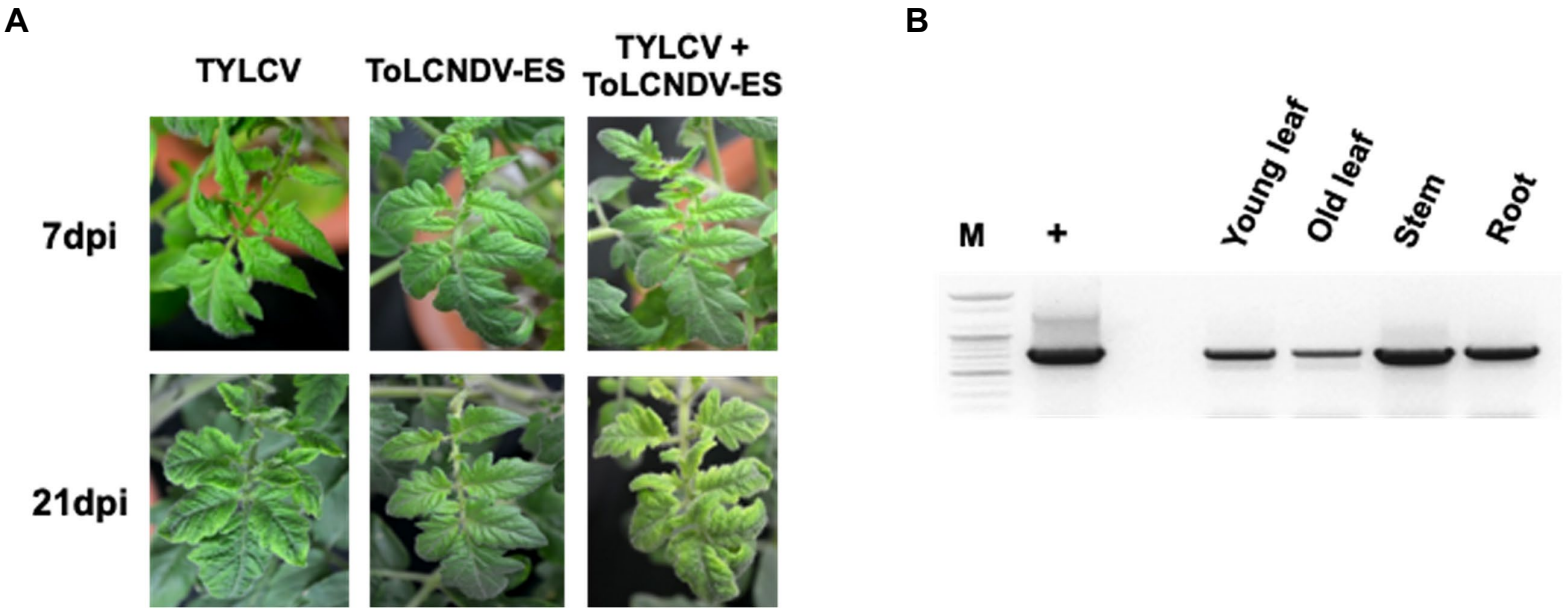
ToLCNDV appearance on co-inoculated tomato plants (San Pedro cultivar): **(A)** phenotype of single and co-inoculated tomato at 7 and 21 dpi. Leaf yellowing symptom was induced in TYLCV and co-inoculated plants, whereas ToLCNDV-ES inoculation displayed a normal phenotype. The symptom severity in co-infected plants was a little higher than a single TYLCV infection; **(B)** PCR results of ToLCNDV-ES on different organs including young leaf, old leaf, stem, and root of tomato. Lane M: ladder, lane +: positive control, lane −: negative control. DNA A of this virus was found in all tested tissues.

The infectivity of single ToLCNDV and TYLCV clone were assessed by PCR and symptom observation after 3 weeks from agro-inoculation. All the tomato plants agroinoculated with TYLCV were positive in PCR and, with the exception of the TYLCV-resistant cultivar Bacchus, developed leaf curling phenotype. No infection was found on tomatoes inoculated with ToLCNDV-ES alone (Table 2; Supplementary Figure S1). The same scenario was observed in the plants simultaneously inoculated with infectious clones of ToLCNDV-ES DNA A/B and TYLCV, where only the TYLCV-resistant cultivar Bacchus displayed a normal phenotype compared to control plants, while all the others, including Moneymaker, San Pedro, Roma VF, San Marzano and Sorrento, displayed leaf yellowing at 21 dpi in 10–50% of the plants, depending on the cultivar (Table 3). The San Pedro cultivar showed the highest infection rate (50%) of ToLCNDV-ES, whereas the other cultivars showed a lower infection ratio, ranging between 10 to 30% of co-inoculated plants. Moreover, symptoms severity in co-infected plants of the San Pedro cultivar were slightly higher than in plants with single TYLCV infection (Figure 4A). The Principe Borghese was the only tomato cultivar not infected by ToLCNDV-ES when co-inoculated with TYLCV. In ToLCNDV-ES positive plants, the PCR results showed the presence of the virus in different organs (including leaves, roots and stems) of each cultivar (Figure 4B). These results suggest that ToLCNDV-ES can replicate and move to other tissues inside co-infected tomatoes.

**Table 2:** Infectivity of single infections with ToLCNDV and TYLCV infectious clones on tomato.

Infectious clone	Cultivars	Infectivity	PCR	Symptoms
ToLCNDV	Moneymaker	0/9^*^	−	−
	Seogwang	0/9	−	−
	San Pedro	0/9	−	−
TYLCV	Moneymaker	9/9	+	Leaf curling, yellowing
	Seogwang	9/9	+	Leaf curling, yellowing
	San Pedro	9/9	+	Leaf curling, yellowing

* Number of infected plants/number of agroinoculated plants.

**Table 3:** Infectivity of ToLCNDV co-inoculated with TYLCV infectious clone on different tomato *via* PCR amplification and symptom observation at 21 dpi.

Tomato cultivars	Infectivity^*^			Symptoms
	TYLCV	ToLCNDV DNA A	ToLCNDV DNA B	
Bacchus^**^	9/10	0/10	0/10	No symptom
Seogwang	10/10	1/10	0/10	Leaf yellowing
Moneymaker	10/10	1/10	0/10	Leaf yellowing
San Pedro	10/10	5/10	0/10	Leaf yellowing
Roma VF	10/10	1/10	0/10	Leaf yellowing
Principe Borghese	10/10	0/10	0/10	Leaf yellowing
San Marzano	10/10	1/10	0/10	Leaf yellowing
Sorrento	10/10	3/10	0/10	Leaf yellowing

* Number of infected plants/number of agroinoculated plants.

** Reported as resistant to TYLCV mediated by the *Ty-1* gene.

### ToLCNDV detection on TYLCV susceptible and resistant tomato

**Figure 5:**
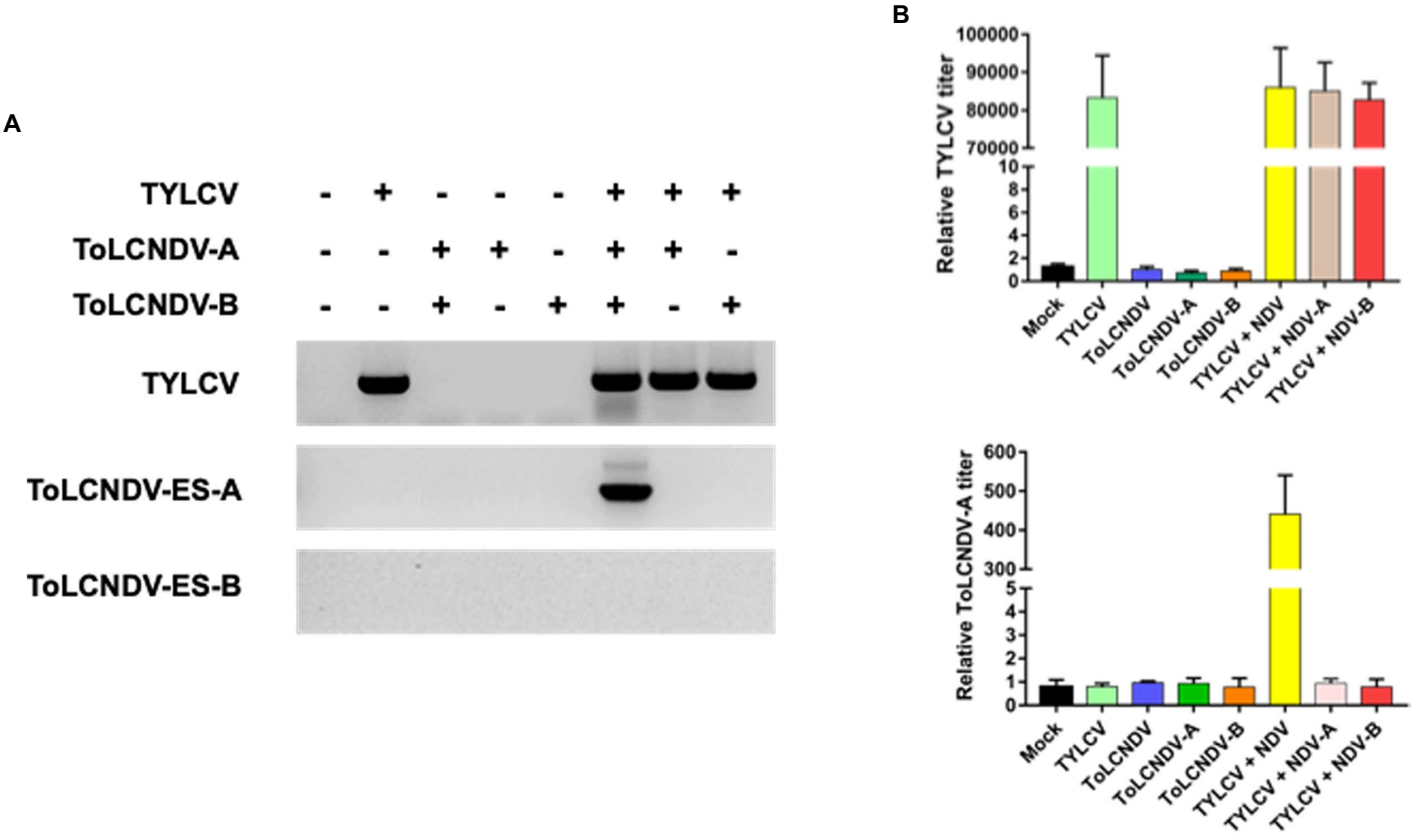
Co-inoculation result on TYLCV susceptible tomato (San Pedro cultivar): **(A)** PCR amplification results between TYLCV and ToLCNDV-ES component after 21 dpi. Seven mixed infections were tested at the same time under the presence (+) or absence (−) of different viral components. Only co-inoculated plants were positive for ToLCNDV-ES presence; **(B)** qPCR result for relative TYLCV and ToLCNDV-ES titers on co-inoculated plants. Titer in mock plants was normalized to 1. Relative viral titers were calculated by the 2^−ΔΔCt^ method. Statistical analysis by *t*-test (*p* value < 0.05) indicated no significant difference in TYLCV amount among single and mixed infection plants, whereas the ToLCNDV-ES titer in co-infected tomato showed a significant difference.

**Figure 6:**
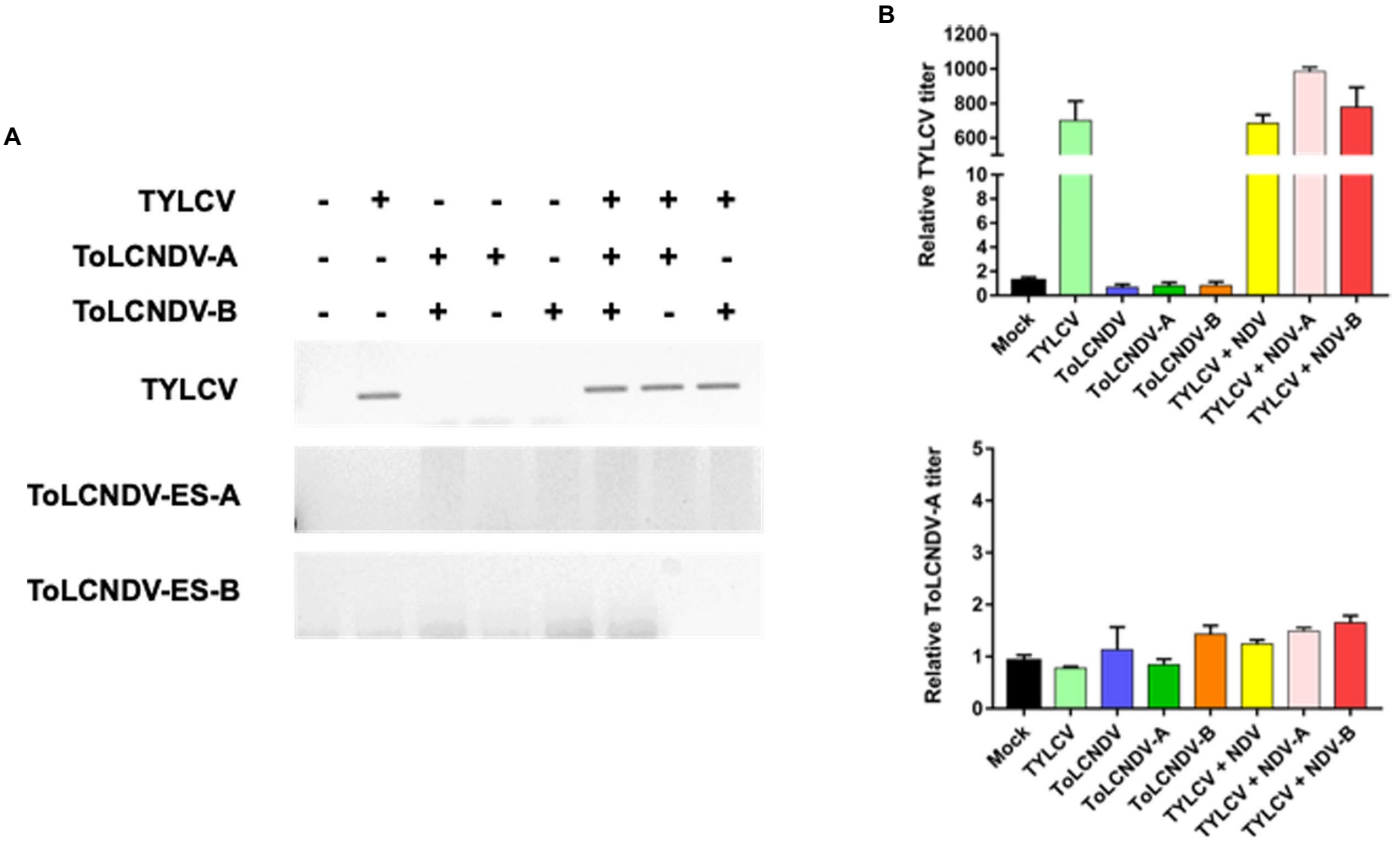
Mixed inoculation of TYLCV and ToLCNDV on TYLCV resistance tomato cultivar “Bacchus”: **(A)** detection of the virus by PCR. In the resistance plants, TYLCV accumulation was reduced and no ToLCNDV was found in the association with the presence (+) or absence (−) of TYLCV; **(B)** relative TYLCV and ToLCNDV amounts on co-inoculated plants were conducted *via* qPCR. Titer in mock plants was normalized to 1. Co-inoculation between TYLCV and ToLCNDV-ES did not result in a significant change in the TYLCV titer compared to a single infection.

To determine whether TYLCV enabled ToLCNDV-ES infection in tomato plants, mixed infections were applied to TYLCV-susceptible and resistant cultivars (i.e., San Pedro and Bacchus respectively). A single inoculation of TYLCV, ToLCNDV-ES DNA A and/or DNA B served as controls for comparison with the double infection test. After 3 weeks, in the susceptible tomato cultivar., TYLCV was detected by PCR in plants inoculated singly with TYLCV and in those inoculated with TYLCV and ToLCNDV-ES with both DNA or with each single DNA components (Figure 5A). Instead, in the same susceptible cultivar., ToLCNDV-ES DNA A was detected by PCR only in the plants, while the other tests showed the absence of ToLCNDV (Figure 5A). In addition, qPCR data indicated similar results (Figure 5B). The TYLCV titer was not different among infected plants (average relative TYLCV titer: 85.000), while the highest ToLCNDV-ES DNA A titer was recorded in the TYLCV/ToLCNDV-ES A-B condition (average relative ToLCNDV-ES titer: 450; Figure 5B). However, DNA B was not detected in any of the tested plants. The commercial TYLCV resistant cultivar showed weak infection of TYLCV and the absence of ToLCNDV-ES in all cases by PCR (Figure 6A) and qPCR (Figure 6B) analysis.

### ToLCNDV infectivity with other begomoviruses in tomato

**Figure 7:**
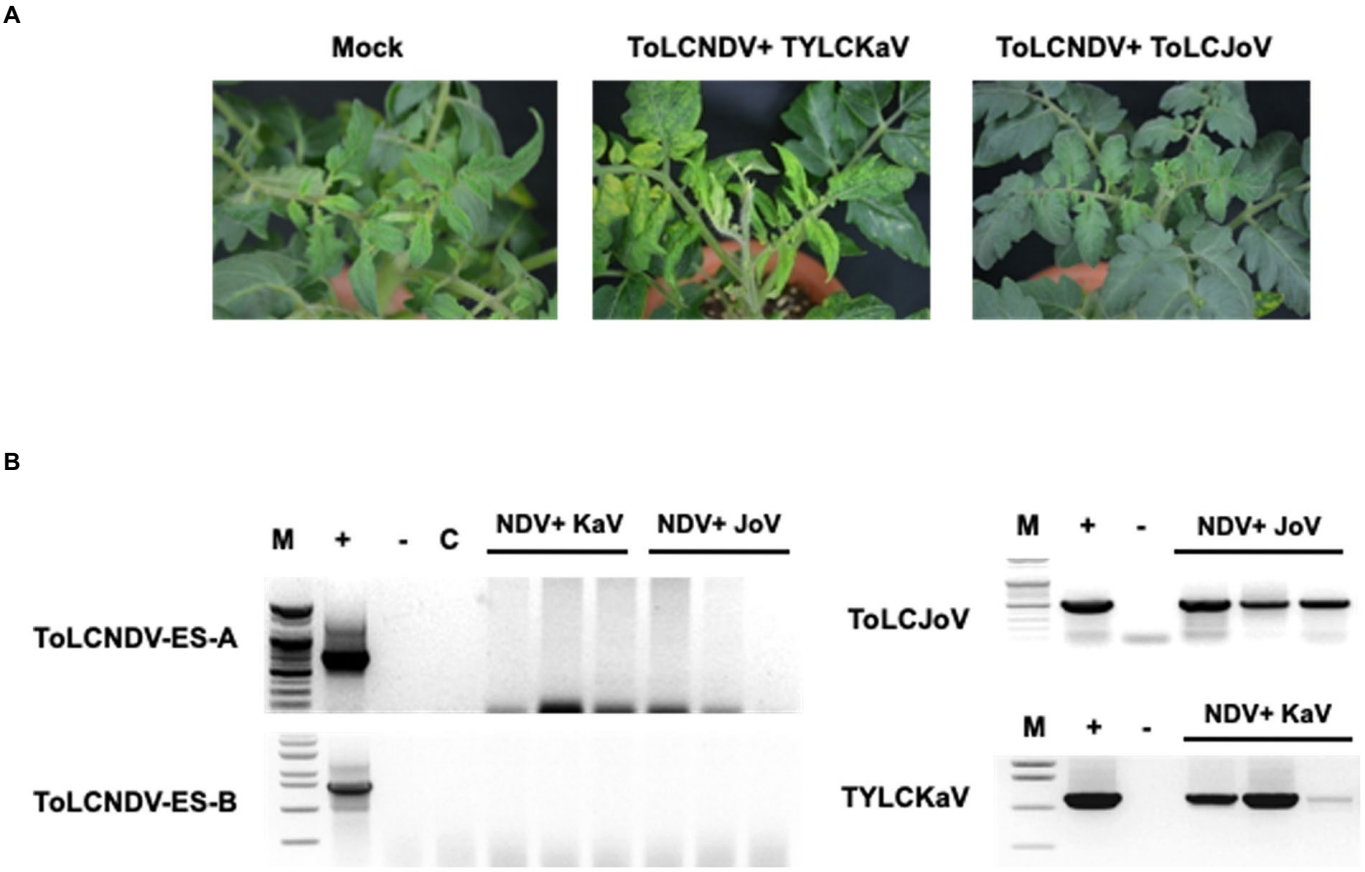
Results for co-inoculation between ToLCNDV-ES and other virus clones in tomato: **(A)** phenotype of San Pedro cv. plant after co-inoculation for 3 weeks. Viral phenotype was induced in mixed infection between TYLCKaV and ToLCNDV-ES. Combination with ToLCJoV showed asymptomatic phenotype compared to mock plants; **(B)** detection result by PCR amplification. Lane M: ladder, lane +: positive control, lane −: negative control, lane C: mock plant. All plants displayed no infectivity of ToLCNDV-ES even mixed with TYLCKaV or ToLCJoV. Infected plants showed only TYLCKaV and ToLCJoV infection.

To check whether other begomoviruses, such as TYLCV, can also enable ToLCNDV-ES to infect tomato, a combination of ToLCNDV-ES and TYLCKaV and ToLCJoV was applied to the San Pedro cultivar. The results at 21 dpi showed no ToLCNDV-ES infection when co-inoculated with other begomoviruses. Plants exhibited yellow and leaf curl phenotypes under TYLCKaV co-infection but normal phenotypes under ToLCJoV (Figure 7A). These phenotypes were similar to those of single-infected plants. PCR results only showed TYLCKaV or ToLCJoV specific amplicons, while no amplicon was observed for ToLCNDV-ES (Figure 7B). These results show that not all begomoviruses are able to complement and help ToLCNDV-ES infect tomatoes.

## Discussion

Whitefly transmitted viruses are emerging diseases of important food and fiber crops (Navas-Castillo et al., 2011). ToLCNDV is an economically important begomovirus transmitted by the whitefly *B. tabaci* and recently emerged in the Mediterranean area where it is causing major damage mainly in cucurbits (Fortes et al., 2016; Panno et al., 2016, 2019; Parrella et al., 2018). The Mediterranean ToLCNDV sequences were found to be genetically distant from the Asian strain and were classified as a different strain (ToLCNDV-ES). The two phylogenetically distantly groups of ToLCNDV isolates differ also for their pathogenicity in tomato and cucurbit crops, with ToLCNDV Asian isolates able to replicate efficiently in tomato, causing a clear viral disease syndrome, while ToLCNDV-ES isolates mainly adapted to infect cucurbits (Yamamoto et al., 2021).

ToLCNDV-ES caused yield losses of up to 20% in melon and 22% in zucchini crops in Almeria, Spain (Sáez et al., 2017; Crespo et al., 2020). In southern Italy, high incidences of disease (80–100%) have been reported in *C. moschata* crops in open fields (Parrella et al., 2018). Thus, ToLCNDV-ES has been listed in the Mediterranean region as a serious threat to cucurbit crops but not to tomatoes, since no significant outbreak of this virus has been recorded in tomato crops in this geographic area. In particular, ToLCNDV-ES was never detected in tomato in Italy so far. However, research regarding this pathogen characteristic should be continuously conducted to prevent future damage in non-permissive plants.

In this study, the infectious clone of ToLCNDV-ES isolated from Italy, belonging to subgroup I of ToLCNDV Italian isolates, showed no infectivity in tomatoes and coincided with the field observation data collected so far, since the first report of ToLCNDV in continental Italy (Parrella et al., 2018). Although the artificial infectious clone of the ToLCNDV-ES isolate used in our study showed a minor difference from previous reports of ToLCNDV identified in Spain (Fortes et al., 2016; Yamamoto et al., 2021), all the data obtained in the present study showed that tomatoes are non-permissive hosts for the subgroup I ToLCNDV-ES Italian isolate. Moreover, some results indicated that when TYLCV was impaired, ToLCNDV-ES could not infect tomatoes (see section “Coinoculation of TYLCV and ToLCNDV infection clones” and Figure 5).

Overall, data obtained from field and agroinoculation tests suggested also a possible different infectivity in tomato between subgroup I and subgroup II of Italian ToLCNDV isolates, with subgroup I unable to infect tomato even at subliminal mode. Similar results were also obtained in a previous study, concerning the different infectivity in cucurbits between the same ToLCNDV-ES Italian isolate and a ToLCNDV Pakistani isolate (Vo et al., 2022). Interestingly, the Italian subgroup I is phylogenetically more distantly related to the ToLCNDV Asian strain than the Italian subgroup II of ToLCNDV isolates (Panno et al., 2019).

During field surveys, we found that tomato plants infected with ToLCNDV-ES (about 50% of symptomatic plants) correlate strictly with simultaneous presence of TYLCV, suggesting that TYLCV may enhance ToLCNDV infection in tomato (Table 1 and Figure 1). In 2014, Simón et al. (2017) conducted a field survey in southern Spain for tomato begomovirus infection by sampling 50 tomato plants showing tomato leaf curl disease (TYLCD). They found 41% of the plants infected by ToLCNDV-ES in double or complex infection with other begomoviruses, such as TYLCV, TYLCSV and tomato yellow leaf curl Axarquia virus (TYLCAxV). As observed in our experiments of agroinoculation, mixed infections of the tomato observed in southern Spain plants correlate with more severe symptoms than plants with single infection (Simón et al., 2017). Nevertheless, differently from our results, during field survey they found tomato plants infected by ToLCNDV, although in low percentage. This apparently difference between the results obtain after field survey in Spain and Italy could be explained considering that ToLCNDV-ES field isolate from Spain and those from Italy, in particular from Campania and Lazio regions (central Italy), belongs to two different subgroups of ToLCNDV isolates, with isolates belonging to subgroup I (from Campania and Lazio) which evolved more recently in south-central continental Italy, probably specializing prevalently in infecting cucurbits (Panno et al., 2019). Interestingly, during our survey we found that the *B. tabaci* genotype associated to TYLCD syndrome in tomato, belonged to the Q2 mitotype of the MED genotype. This finding is in complete agreement with previous results demonstrating, as results of an extensive survey on *B. tabaci* populations conducted in the same Italian region, a near complete displacement of the Q1 by the Q2 mitotype, especially in protected solanaceous crops (Parrella et al., 2014). Moreover, the displacement of the Q1 mitotype of *B. tabaci* correlated also with the disappearance of TYLCSV (Parrella et al., 2014). Thus, the data produced in the present work confirmed preliminary observation on the *B. tabaci* genotype and geminiviruses spreading in Campania region in solanaceous crops. Nevertheless, it must be considered that this scenario is different from that described in other countries of the Mediterranean basin. In Spain, the Q1 mitotype of *B. tabaci* is prevalent in the field and protected crops and, interestingly, also TYLCSV is routinely detected in tomato cultivations together with TYLCV, TYLCAxV and more recently with ToLCNDV (Moriones et al., 2017; Simón et al., 2017).

To confirm our findings related to 2019–2020 field surveys, experimental agroinoculation of ToLCNDV and TYLCV infectious clones was conducted in various tomato cultivars, and the results were verified with that of the field reports. In addition, disease symptoms in double-infected plants were more severe than those in single infections. This implies that TYLCV and ToLCNDV-ES may have synergistic interactions in tomatoes. Several co-infection studies on the synergistic interaction between begomoviruses have been reported, and their synergism has led to increased symptom severity and significant economic impact of plant diseases. For examples, mix infection of pepper huasteco virus (PHV) and pepper golden mosaic virus (PepGMV) lead to increased their replication in tobacco (Méndez-Lozano et al., 2003). In addition, the two bipartite begomoviruses, PHV and tomato mottle Taino virus (ToMoTV), can complement infections in tomato and also pseudorecombine (Guevara-González et al., 1999).

Synergistic effects have also been observed during the interaction of begomoviruses with other distantly related viruses. The occurrence of TYLCV and tomato chlorosis virus (ToCV) co-infection has been on the rise and has promoted their spread in the field (Martínez-Zubiaur et al., 2008; Dai et al., 2017; Wei et al., 2018). Mixed infection of the Asian ToLCNDV strain with other viruses or satellites has also been reported (Shafiq et al., 2010; Sivalingam and Varma, 2012; Singh et al., 2016; Zaidi et al., 2017). These results imply that the satellite components can replace ToLCNDV DNA B in the movement of DNA A, resulting in more severe symptoms, whereas DNA A alone induced local infection and a mild disease phenotype. Similarly, in tomato plants agroinoculated with both TYLCV and ToLCNDV, only ToLCNDV DNA A was identified by PCR and qPCR in different tissues, including new leaves, shoots, and roots (Figure 3; Supplementary Figure S2), while, after co-inoculation of tomato plants, DNA B disappeared and was no longer detected in different parts of the plant. Such results would suggest that TYLCV favors the movement of ToLCNDV in the tomato plant, probably complementing the function of the movement protein carried by the DNA B. In double-infected plants, the ToLCNDV DNA A component was found in different tissues, but when we examined plants double-infected with TYLCV and others with only ToLCNDV DNA A, no evidence of ToLCNDV infection was observed. These results suggest that TYLCV proteins may assist ToLCNDV-ES DNA A replication as well as the replacement of the DNA B function of virus movement into the host, allowing ToLCNDV-ES to invade non-directly inoculated tomato plants tissues.

To our knowledge, this is the first report on the functional complementation and synergistic interaction between ToLCNDV-ES and other begomoviruses in the Mediterranean region. The ToLCNDV-ES Italian isolate in this study was only detected in double infection with TYLCV, which is associated with both components of ToLCNDV in the primary infection. Nevertheless, not all begomoviruses enhance ToLCNDV as TYLCV in mixed infections. We even tested co-infection with other monopartite (ToLCJoV) and bipartite (TYLCKaV) begomoviruses but neither of them had any positive effect on ToLCNDV infection. Thus, the synergistic relationship with TYLCV may play a key role in understanding the low or absent ToLCNDV-ES infectivity on tomato.

The complementation and synergistic effects in tomato caused by the double infection of ToLCNDV-ES and TYLCV can represent a new problem for tomato crops due to the following reasons: (a) in many countries of the southern Mediterranean area, two begomoviruses transmitted by the same vector are often present in the same cultivation areas of cucurbits and tomatoes; (b) symptoms observed on tomato plants by ToLCNDV-ES and TYLCV together were more severe than those induced by TYLCV alone; (c) there is always a risk that new recombinants with unknown phenotypes will be formed between two begomoviruses or a new viral species will be selected over time.

For these reasons, it is necessary to continue the monitoring and identification of viruses in the areas most at risk (e.g., where the two viruses coexist) and to adopt, when possible, some control measures such as the use of tomato genotypes that are resistant to TYLCV or ToLCNDV, in order to reduce the risk of selecting new recombinants from plants harboring mixed infections.

## Conclusion

Our study provides a new finding on ToLCNDV-ES infection associated with TYLCV in tomatoes. The data reported from field and laboratory conditions demonstrate that the incidence of infection of a ToLCNDV-ES Italian isolate increases in case of contemporary infection with TYLCV as compared to single infections. Nevertheless, not all begomoviruses can enhance ToLCNDV-ES infection in tomato. Although further studies are needed to determine the molecular mechanisms that regulate ToLCNDV-ES infection in tomatoes, this finding highlights the risk of ToLCNDV-ES based on its increased pathogenicity in non-permissive hosts in combination with TYLCV, since both viruses are spreading or are already present in several Mediterranean countries.

## Data availability statement

The original contributions presented in the study are included in the article/Supplementary material, further inquiries can be directed to the corresponding author.

## Author contributions

GP conceived and developed the concept, supervised the experiments, and wrote the manuscript. TV, AL, PH, E-JK, and SL contributed to the data analysis and interpretation, discussions, and writing of the manuscript. TV, ET, and GP performed the experiments. All authors contributed to the article and approved the submitted version.

## Funding

This work was supported under the framework of international cooperation program as part of a bilateral project managed by the National Research Foundation of Korea and the National Research Council of Italy (2017K2A9A1A06035325) and by the Campania Region-funded URCoFi project “Strengthening of the supervision activities and control of pests.”

## Conflict of interest

The authors declare that the research was conducted in the absence of any commercial or financial relationships that could be construed as a potential conflict of interest.

## Publisher’s note

All claims expressed in this article are solely those of the authors and do not necessarily represent those of their affiliated organizations, or those of the publisher, the editors and the reviewers. Any product that may be evaluated in this article, or claim that may be made by its manufacturer, is not guaranteed or endorsed by the publisher.
