# Supplementary material for: ToLCNDV-ES infection in tomato is enhanced by TYLCV: Evidence from field survey and agroinoculation
Source: Front Microbiol. 2022 Nov 8;13:954460. doi: 10.3389/fmicb.2022.954460 (PMC9679516; doi:10.3389/fmicb.2022.954460)
Supplement: Supplementary file 1 [file Table_1.docx]

**Table S1: Primer set used in this study**

| **Purpose** | **Primer name** | **Primer sequence 5’-3’** | **Target size** |
| --- | --- | --- | --- |
| **Detection** | ToLCNDV-A-detect-F | AAGCTTAAAACGTGTCGTTTCGATCTGG | 725 bp |
|  | ToLCNDV-A-detect-R | CTCGAGTAACATCACTAACACAC |  |
|  | ToLCNDV-B-detect-F | GGTACCCTTAACGATCTTGAAC | 1586 bp |
|  | ToLCNDV-B-detect-R | ACTAGTCTACAAAAGATAACGAATGGCAAAT |  |
|  | TYLCV-detect-F | GATGGCCGCGCCTTTTCCTTTTATGTGG | 390 bp |
|  | TYLCV-detect-R | GCTGCTGTATGGGCTGTCGAAGTTCAG |  |
|  | ToLCJoV-detect-F | GAAGTCCGGATGTTCCAAGG | 547 bp |
|  | ToLCJoV-detect-R | GCATACACAGGGTTAGAGGC |  |
|  | TYLKaV-detect-F | GTGGGATCCATTGCTACACC | 1000 bp |
|  | TYLCKaV-detect-R | GGCGTTGGGGATTCACAAG |  |
| **Real-time PCR** | qPCR-ToLCNDV-A-F | TTGCTTTGCCAGTCACGTTG | 175 bp |
|  | qPCR-ToLCNDV-A-R | GCAAAACAATGTGGGCTCGT |  |
|  | qPCR-TYLCV-F | GCTCGTAGAGGGTGACGAAG | 165 bp |
|  | qPCR-TYLCV-R | ACACAAAGTACGGGAAGCCCAT |  |
| **Infection clone construction** | TY1-IC-F1-SalI | GTCGACGTTGAAATGAATCGGTGTCCCTC | 1640 bp |
|  | TY1-IC-R1-SphI | GCATGCGTACATGCCATATACA |  |
|  | TY1-IC-F2-SphI | GCATGCCTCTAATCCAGTGTAT | 1407 bp |
|  | TY1-IC-R2-BglII | AGATCTATTGCAAGACAAAAAACTTGGGGAC |  |
