# Supplementary material for: ToLCNDV-ES infection in tomato is enhanced by TYLCV: Evidence from field survey and agroinoculation
Source: Front Microbiol. 2022 Nov 8;13:954460. doi: 10.3389/fmicb.2022.954460 (PMC9679516; doi:10.3389/fmicb.2022.954460)

Figure S1 - Infectivity results of single ToLCNDV-ES infection on different tomato cultivars/line: (A) Phenotype of inoculated plants at 21 dpi; (B) PCR results at 21 dpi.

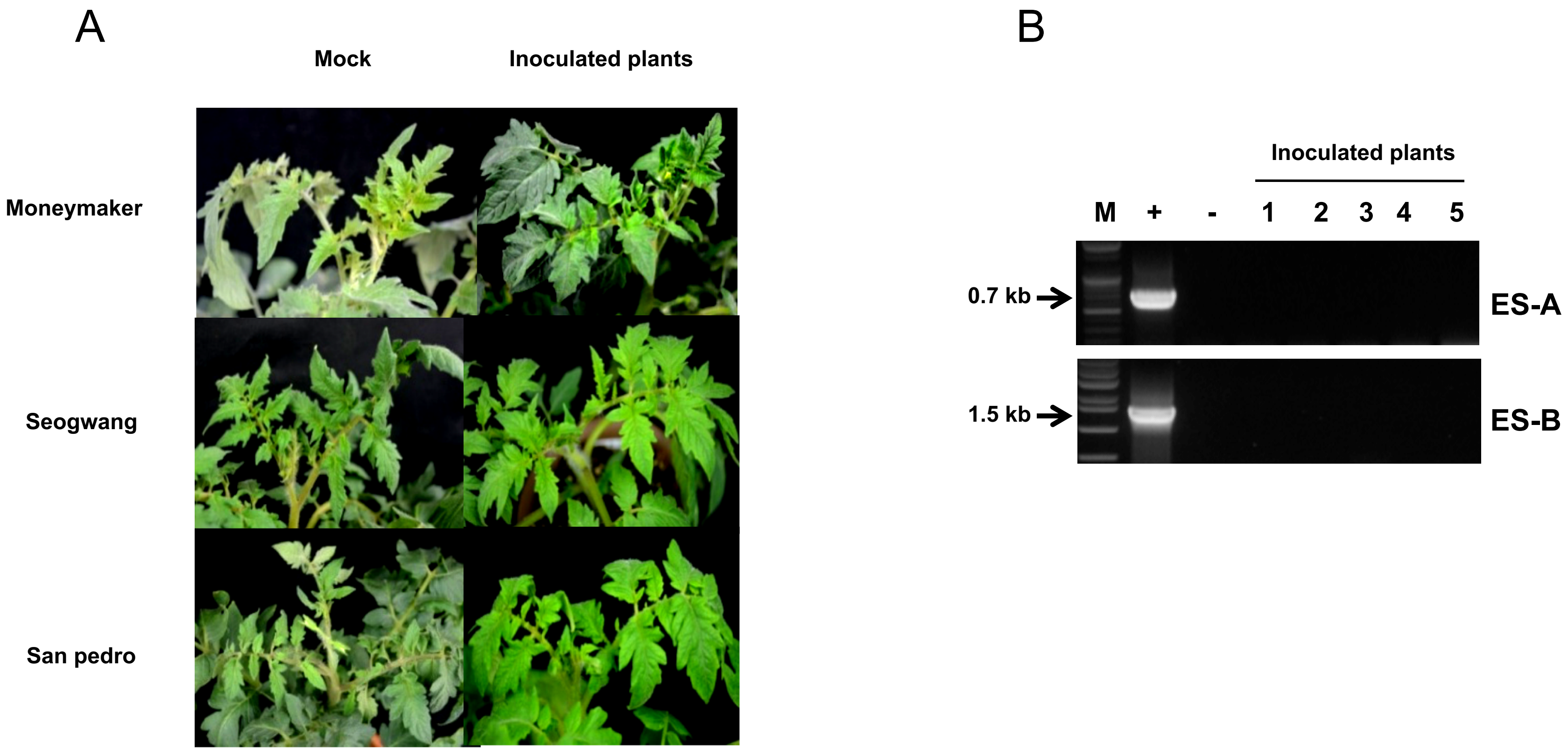

Supplement: Supplementary file 2 [file Image_1.pdf]
