# Supplementary material for: ToLCNDV-ES infection in tomato is enhanced by TYLCV: Evidence from field survey and agroinoculation
Source: Front Microbiol. 2022 Nov 8;13:954460. doi: 10.3389/fmicb.2022.954460 (PMC9679516; doi:10.3389/fmicb.2022.954460)

Figure S2 - Relative ToLCNDV DNA A titer in co-inoculated plants (TYLCV and ToLCNDV-ES) of San Pedro cultivar detected at 7, 14 and 21 dpi by qPCR.

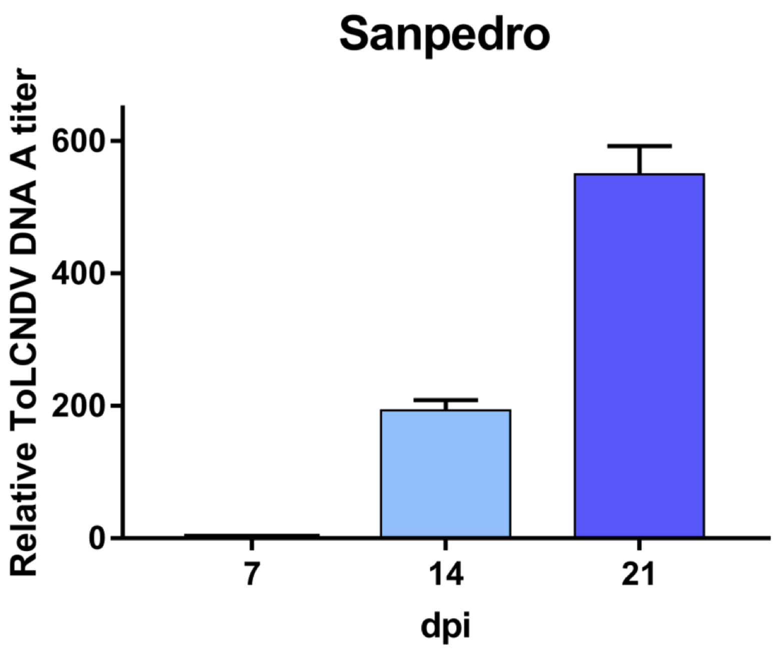

Supplement: Supplementary file 3 [file Image_2.pdf]
